# Children Use Non-referential Gestures in Narrative Speech to Mark Discourse Elements Which Update Common Ground

**DOI:** 10.3389/fpsyg.2021.661339

**Published:** 2022-01-11

**Authors:** Patrick Louis Rohrer, Júlia Florit-Pons, Ingrid Vilà-Giménez, Pilar Prieto

**Affiliations:** ^1^Grup d’Estudis de Prosòdia (GrEP), Department of Translation and Language Sciences, Universitat Pompeu Fabra, Barcelona, Spain; ^2^Université de Nantes, UMR 6310, Laboratoire de Linguistique de Nantes (LLING), Nantes, France; ^3^Department of Subject-Specific Education, Universitat de Girona, Girona, Spain; ^4^Institució Catalana de Recerca i Estudis Avançats (ICREA), Barcelona, Spain

**Keywords:** information structure (IS), discourse referents, referential gesture, non-referential gesture, multimodal development, narrative discourse, child development

## Abstract

While recent studies have claimed that non-referential gestures (i.e., gestures that do not visually represent any semantic content in speech) are used to mark discourse-new and/or -accessible referents and focused information in adult speech, to our knowledge, no prior investigation has studied the relationship between information structure (IS) and gesture referentiality in children’s narrative speech from a developmental perspective. A longitudinal database consisting of 332 narratives performed by 83 children at two different time points in development was coded for IS and gesture referentiality (i.e., referential and non-referential gestures). Results revealed that at both time points, both referential and non-referential gestures were produced more with information that moves discourse forward (i.e., focus) and predication (i.e., comment) rather than topical or background information. Further, at 7–9 years of age, children tended to use more non-referential gestures to mark focus and comment constituents than referential gestures. In terms of the marking of the newness of discourse referents, non-referential gestures already seem to play a key role at 5–6 years old, whereas referential gestures did not show any patterns. This relationship was even stronger at 7–9 years old. All in all, our findings offer supporting evidence that in contrast with referential gestures, non-referential gestures have been found to play a key role in marking IS, and that the development of this relationship solidifies at a period in development that coincides with a spurt in non-referential gesture production.

## Introduction

In face-to-face communication, people naturally use gestures while speaking. Co-speech gestures have been defined as visible actions made by bodily movements (hands, head movements, among others) that act as an utterance or a part of an utterance and co-occur with speech (Kendon, 2004; see Wagner et al., 2014, for a review). Particularly, manual co-speech gestures are strongly connected to speech from three different perspectives: semantics, pragmatics, and phonology (Kendon, 1980; McNeill, 1992). Indeed, according to McNeill’s (1985; 1992; 2005) three “synchrony rules,” gestures are co-expressive with the semantic and pragmatic meaning expressed in speech, and prominent movements in gesture (i.e., the stroke) occur just before or concurrently with stressed syllables in speech.

**FIGURE 1:**
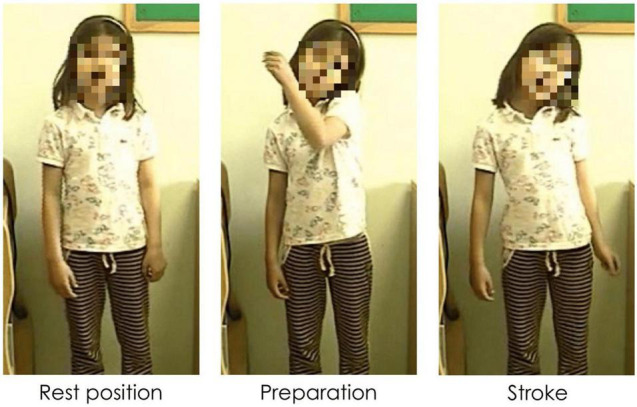
Caption of an iconic gesture while saying “va DESFER la corda” (“[he] UNTIED the rope”). Capitalization indicates the word accompanied by a gesture.

**FIGURE 2:**
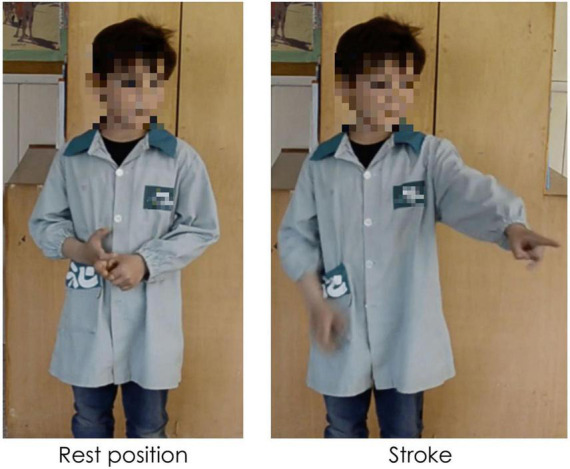
Caption of a deictic gesture while saying “aquell RATOLÍ” (“that MOUSE”). Capitalization indicates the word accompanied by a gesture.

**FIGURE 3:**
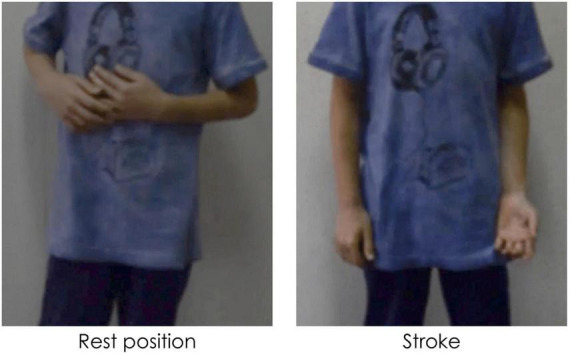
Caption of a non-referential gesture while saying “hi havia el MATEIX ANIMAL” (“there was the SAME ANIMAL”). Capitalization indicates the word accompanied by a gesture.

Gestures can be distinguished in terms of their referentiality. *Referential gestures* can be iconic or metaphoric in nature through using “pictorial” hand shapes or movements to describe concrete or abstract entities and actions described in speech, or deictic in nature through locating concrete or abstract entities in space. These gestures clearly and directly represent the semantic content of speech, adding to the propositional meaning of the utterance. *Non-referential gestures*, often referred to as “beat” gestures, have been traditionally described as rhythmic and prominence markers. However, we use a recent and more comprehensive definition that does not limit these gestures to “a simple flick up-and-down or in-and-out,” (McNeill, 1992, p. 15). Indeed, these gestures can have more complex gesture phasing, forms, and tend to have discursive and pragmatic functions (Shattuck-Hufnagel et al., 2016; Prieto et al., 2018; Shattuck-Hufnagel and Prieto, 2019; Rohrer et al., 2020; among others). We consider these gestures as non-referential in nature as they do not portray any semantic meaning in speech (*via* iconicity, metaphoricity, or deixis) through its hand shape or trajectory movement. Figures 1–3 show examples of referential (e.g., iconic and deictic) and non-referential gestures.

With respect to language development, research has shown that gesture and speech develop in parallel. In fact, children start producing referential (deictic) gestures to refer to an object, people, events, or locations before their first birthday (Bates, 1976; Bates et al., 1979) and these are produced along with one-word vocalizations at the age of 11 months to one-and-a-half years (e.g., Butcher and Goldin-Meadow, 2000; Goldin-Meadow and Butcher, 2003; Murillo and Belinchón, 2012; Esteve-Gibert and Prieto, 2014). Shortly after development (e.g., by 26 months, see Özçalışkan and Goldin-Meadow, 2011), there is a spurt in the production of referential (iconic) gestures. It is not until around the age of 2–3 that children begin producing non-referential gestures in spontaneous interactions, often, to make speech more emphatic (see Nicoladis et al., 1999; Levy and McNeill, 2013). It is at around the ages of 4–6 that non-referential gestures start to be employed in children’s more complex and elaborated discourses, such as narratives (Graziano, 2009; Colletta et al., 2010, 2015; Mathew et al., 2018; Florit-Pons et al., 2020). Importantly, existing research has demonstrated that referential and, particularly, non-referential gesture use develops in parallel to discursive skills (Graziano, 2009; Colletta et al., 2010, 2015; Florit-Pons et al., 2020). However, it remains unclear how the use of referential and non-referential gestures is linked to information structure (henceforth, IS) in children’s narrative discourse, and how this relationship develops longitudinally.

The present paper focuses on the multimodal development of children’s narrative discourse by specifically asking how both referential and non-referential gestures are related to IS-marking^1^. We believe that it is crucial to investigate the role of non-referential gestures and their contrast to referential gestures in IS-marking, specifically because previous literature has defined non-referential gestures as being special discourse pragmatic markers (see Section “Relationship Between Gesture and Information Structure”). Given that previous studies found a significant increase in non-referential gesture production while discourse skills develop in children, there is a clear need to investigate the discourse pragmatic dimension of these two types of gestures so that we have a more thorough understanding of multimodal development.

### Information Structure

Information Structure can be broadly described as the ways in which speakers “package information” to transmit to their interlocutors, with the goal of updating the shared common knowledge between speakers (i.e., their common ground) and ultimately move communication forward (e.g., Chafe, 1976; Krifka, 2008). However, studies regarding IS, in general, have used a variety of approaches, often employing imprecise or overlapping terms (see, e.g., Lambrecht, 1994; Krifka, 2008, among others).

The current study follows theoretical work by Skopeteas et al. (2006) and the Linguistic Information Structure Annotation (LISA) guidelines (Götze et al., 2007; Ritz et al., 2008) which identifies three independent, non-mutually exclusive levels of IS. The most general of these levels is the level of Focus/Background where a two-way distinction identifies focus as parts of the utterance that are most relevant to discourse and function to move the discourse forward. Background, thus, refers to the other parts of the utterance that do not serve to develop the discourse (e.g., irrelevant or repeated information). In terms of narrative productions, the text-initial clause is almost always all focus.

This two-way distinction of what is moving the discourse forward and what is not can then also be analyzed at a second level of IS, that of Topic/Comment. The level of Topic/Comment corresponds to the Prague School’s Theme/Rheme distinction (Sgall et al., 1986, as cited in Krifka, 2008), where we can identify “aboutness” topics as referents upon which the remainder of the sentence is predicated. Topics may also be considered “frame-setting” in that they offer the interpretive frame for the predication. The comment is thus the predication, itself, that is made about the topic. Sentences that only contain an action or answer the question “What happened?” or “What’s happening?” are considered topic-less sentences (i.e., all-comment).

**FIGURE 4:**
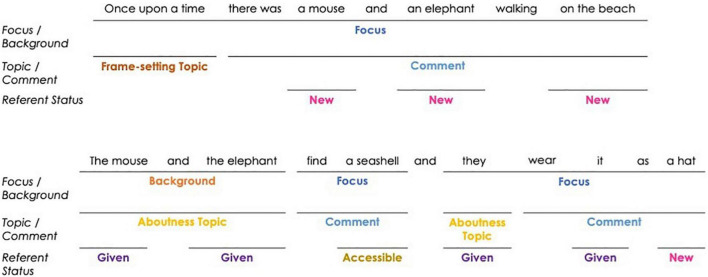
The information structure (IS) of a child’s narrative. Please see the Supplementary Appendix to see a complete example of the Gesture/IS coding and for more examples of each category.

The levels of Focus/Background and Topic/Comment differ in that the former considers what novel information is being uttered to update and move discourse forward (focus) or not (background), while the latter, the thematic content of the utterance (“what is the topic of discussion”) and its predication (“what is being said about that topic”) are considered. While there may be a certain amount of correspondence between topic/background constituents and comment/focus (e.g., the second sentence in Figure 4), topics can also be a part of focus constituents, particularly when they are in the subject position of the utterance which is moving the discourse forward (see the second clause of the second sentence in Figure 4). Following LISA guidelines, these two levels are annotated in an independent fashion, with no presupposed relationship between them (e.g., see Ritz et al., 2008, p. 2138).

The most precise of the IS levels is the categorization of the information status of individual discourse referents (henceforth, Referent Status). These include any noun or prepositional phrases that specifically refer to entities in the discourse such as individuals, places, times, and events, which can receive anaphoric expressions. Referents can be identified as being new, accessible, or given, which is based on the degree of cognitive activation of these referents for the addressee. Given referents have been explicitly mentioned in previous discourse and are thus cognitively active. Slightly less cognitively active referents are said to be accessible, in that they can be inferred from context or are assumed to be familiar through cultural or world knowledge. These can also include unique referents, such as *the Sun* or *Barcelona*. New referents are those which have not been used in context and are cognitively inactive for listeners (i.e., they cannot be inferred from context).

Figure 4 below shows the IS annotation of a child’s brief discourse which contains examples of the different IS labels at all three levels (also refer to the Supplementary Appendix for different examples of IS categories and for a full annotation of a child’s narration). Again, it is important to reiterate that these different levels of IS are not mutually exclusive. Referents may be found in either topic or comment constituents regardless of their referent status, and specifically given referents (regardless of their Topic/Comment status) can be found in either background or focus constituents (e.g., “they” in Figure 4, as it is a given referent that is updating the common ground in terms of who is wearing the hat). The introduction of new referents, by default, are functioning to update the discourse and are thus always in focus constituents (though they may be either topic or comment).

### Relationship Between Gesture and Information Structure

Based on theories of Communicative Dynamism (CD; i.e., referring to the variation in the communicative importance of the different constituents in a sentence) and in line with Givón’s (1983) Principle of Quantity (see also the theory of Effort Code by Gussenhoven, 2002), McNeill (1992, 2005) also suggested that both referential and non-referential gestures are more likely to co-occur with less readily accessible information (i.e., newer information that propels the discourse forward). According to McNeill, a narrator’s gesture can indeed be a window onto which elements of a story are the most crucial for advancing the story based on the gestural output, and when there is little CD, speech tends to not be accompanied by a gesture. Specifically, the author predicts a progression as a function of increasing CD, where more thematic elements (i.e., topics) are said to have a very low CD and thus, often do not co-occur with a gesture. As CD increases (and speech is more rhematic in nature, commenting on the topic), gesture complexity increases. In other words, it is believed that non-referential and pointing gestures will accompany speech with lower CD, and iconic gestures will accompany points of higher CD, as according to Givón (1985), “less predictable/accessible/continuous a topic is, the more coding material is used to represent it in the language (p. 197, as cited in McNeill, 1992).”

Since then, a number of empirical studies involving adult speech have found that gestures tend to mark the introduction of either new referents (e.g., Marslen-Wilson et al., 1982; Levy and Fowler, 2000; Gullberg, 2003; Yoshioka, 2008; Debreslioska et al., 2013; Debreslioska and Gullberg, 2019) or accessible referents in discourse (Debreslioska and Gullberg, 2020b). Interestingly, Debreslioska et al. (2013) mention that gestures may co-occur with given referents but are more likely to occur when the given referents are reintroduced (i.e., explicitly mentioned in previous discourse, but reactivated in the narrative with a full noun phrase), as opposed to a maintained given referent (i.e., easily accessible to the hearer and produced with a lexically reduced form such as a pronoun, or completely missing, i.e., zero anaphora). Furthermore, Referent Status (as well as clause structure) affect the semantic content that is encoded in referential gestures (see e.g., Foraker, 2011; Debreslioska and Gullberg, 2020a), while subsequent recurrent gestural features (i.e., McNeill’s catchments) may help build cohesion and aid referent tracking (McNeill and Levy, 1993). It is significant to note that all of the previously mentioned studies on gesture marking of Referent Status either do not distinguish between referential and non-referential gestures or have focused exclusively on referential gestures.

Unlike referential gestures, non-referential gestures have been ascribed a number of discourse-pragmatic functions, including adding emphasis, marking focus, introducing new characters, or adding further information (McNeill, 1992, pp. 169-171; see also Kendon, 1980, 1995, 2017; Loehr, 2012; Ferré, 2014; Shattuck-Hufnagel et al., 2016; Prieto et al., 2018; Shattuck-Hufnagel and Prieto, 2019). They are commonly described as a “yellow highlighter,” coupling with prosodic prominence to add emphasis to parts of a discourse that speakers deem important. Even though previous literature has defined non-referential gestures as being discourse pragmatic markers, little empirical evidence has been given to support such claims. In a descriptive study, Loehr (2012) gives examples of how gestures and intonation convey a pragmatic synchrony, such as non-referential gestures coupling with pitch accentuation to add emphasis to speech. Specifically, the author describes how the production of L + H* pitch accents (i.e., a bitonal prosodic prominence with a rising pitch movement during the accented syllable) is much more emphatic and used to mark contrastive elements in a speech to a much larger degree than H* pitch accents (i.e., a monotonal prosodic prominence realized as a high plateau during the accented syllable), and that the latter are often accompanied by non-referential gestures. Another study by Ferré (2014) aimed to understand the interaction between prosody, gesture, and the syntactic marking of focus *via* thematization (i.e., different forms of syntactic fronting, such as moving constituents to the beginning of the sentence) in conversational French. This study is one of the few that includes non-referential gestures and at the same time integrates a thorough description of their conceptualization of focus. The results showed that prosodic, gestural, and syntactic strategies for marking focus were used in a complementary fashion (in so much that speakers generally do not mark focus in all three modes simultaneously). Specifically, Ferré found that non-referential gestures co-occurred with prosodic focus much more than referential gestures, and that (metaphoric) referential gestures co-occurred with syntactic markers of focus more than other gestures.

However, to our knowledge, only one empirical study has investigated the marking of Referent Status focusing specifically on non-referential gestures. Im and Baumann’s (2020) preliminary study investigated the multimodal marking of Referent Status in terms of both prosody and non-referential gesture in a two-and-a-half minute Ted Talk video. Much like Debreslioska and Gullberg (2020b), the study used a precise annotation of referents containing four levels: *new*, *unused* (i.e., unique referents, such as “the Sun”), *bridging* (which corresponds to accessible referents from context), and *given*. Although their study did not carry out any statistical analyses, their results showed a tendency for non-referential gestures to mark more bridging (i.e., accessible), new, and unused referents than given referents.

Fewer studies have investigated the production of gestures specifically in regard to the marking of thematic information (i.e., Topic/Comment). The works by Levy (1984) and Levy and McNeill (1992) describe how gestures often co-occur with the first mentions of discourse referents, which are important for CD as they set up thematic elements for the discourse that follows and ultimately push the discourse forward. They may additionally co-occur with subsequent mentions, particularly when the referent is produced on a different narrative level (used as a “scene-changing device” and affording a separate peak of CD). In this sense, the authors claim that gestures are used for “creating new themes or continuing old ones” (McNeill and Levy, 1993, p. 2).

All in all, while non-referential gestures have been suggested in the literature to be intimately associated with focus-marking and marking of new information, more empirical evidence is needed to confirm this relationship and to compare it to IS-marking with referential gestures. Moreover, very little is known about the IS-gesture referentiality relationship in narrative development.

### Marking of Information Structure in Development

Even though IS can be made apparent through syntactic, morphologic, and lexical means, as well as with prosodic and gestural markers, developmental studies have mostly focused on children’s ability to introduce referents through morphosyntactic means. This concerns whether children are able to introduce new referents with the correct, full morphosyntactic form (e.g., “John went to the shop,” instead of “He went to the shop,” where the listener would not know “he” refers to “John”) or whether they are able to maintain pronominal reference after the initial referent has been introduced (e.g., “My Grandma went to the hospital. *She* was very sick,” taken from Peterson and Dodsworth, 1991, p. 399, italics in original), with most studies finding that mastery of discourse referent introduction and maintenance develops during the school years.

Studies on the development of referent introduction have shown that preschool children are not yet able to introduce new referents adequately. In a longitudinal study which assessed preschoolers’ (2- to 3;6-year-old) narrative development, Peterson and Dodsworth (1991) showed that at the age of 3-and-a-half years, children still produced errors when introducing new referents in discourse, such as using ambiguous referents, incorrect pronouns, or zero anaphora when the referent is not inferable. Though children still produced errors, their use of pronominal referents increased with age along with the mean length of utterance (MLU). These findings were complemented with later results obtained by Guerriero et al. (2006) who analyzed the referential choices for verb arguments in English-speaking and Japanese-speaking children. The authors found, on the one hand, that English-speaking children had difficulties introducing new referents at the age of 1;9 years as they tended to use non-lexical (i.e., null or pronominal) forms instead of lexical forms, but by age 3 no longer made use of non-lexical forms. On the other hand, Japanese children at age 3 were still not able to appropriately use referential forms, indicating that there were some structural differences among languages, which may have an effect on development. Further studies have shown that older children still have some difficulties in IS-marking, and it is not until the ages of 6–7 that they start to master referent introduction (e.g., Hickmann, 1980, 1982; Bamberg, 1986; Kail and Hickmann, 1992; Vion and Colas, 1998). For instance, Vion and Colas (1998) documented that young school-age 7-year-old children began introducing new referents (using definite articles, “the boy” or nouns without articles, “boy”) to the characters in a narrative task, although it was not until later in development (i.e., at the age of 9) that they started to do so in a systematic way and similar to adults, using indefinite articles (see Küntay, 2002, for similar results in Turkish-speaking children; and Schneider and Hayward, 2010, for conflicting results). Interestingly, children’s improvement (from the preschool to school-age years) in referencing discourse entities was found to be linked to their narrative development in that their stories tended to be more comprehensible when they produced fewer errors in referent introduction (Peterson and Dodsworth, 1991; Küntay, 2002).

Developmental studies have also looked at focus-marking, specifically from a prosodic perspective (see, e.g., Chen, 2007, 2010, 2011), showing that prosody helps children mark, and listeners comprehend, focused constituents. A limited amount of research has provided evidence that gestures also help mark focus. For instance, Shattuck-Hufnagel et al. (2016) showed that non-referential gestures produced by 5- to 7-year-old children tended to be temporally aligned with pitch accents, indicating that these gestures were used to mark emphasis or contrastive focus. Esteve-Gibert et al. (2021) showed, however, that French-speaking preschoolers used head nods to mark corrective focus (i.e., an element that “replace[s] a preceding element that was wrongly added to the shared common ground,” p. 2) and that it is easier for them to mark focalized constituents with head gestures when they are located at the end of the prosodic phrase. In a recent study, Koutalidis et al. (2020) showed that 4-year-old German children use non-referential gestures to introduce focus-sensitive particles such as “even,” “also,” and “only” (“noch,” “auch,” and “nur” in German).

Nonetheless, there is still scarce developmental evidence about the role of gesture in the multimodal marking of IS. While research on multimodal focus-marking in adult speech is considerable, how this ability develops in children’s discourse is still unknown. For this reason, the current study aims to see how children use referential and non-referential gestures to mark IS from a longitudinal point of view (e.g., at 5–6 years of age and 2 years later).

### Motivation of the Study: The Role of Non-referential Gestures in Children’s Discourse Development

Previous studies have already investigated the role of referential gestures in the development of children’s narrative discourse (e.g., Demir et al., 2015; Stites and Özçalışkan, 2017). However, recent developmental evidence leads us to believe that non-referential gestures are essential in framing and bootstrapping children’s development of complex narrative discourse abilities. These studies have shown that non-referential (beat) gestures do not only benefit cognitive processes, such as information recall and comprehension (e.g., Austin and Sweller, 2014; Igualada et al., 2017; Llanes-Coromina et al., 2018), but can also act as predictors and causal mechanisms on children’s narrative discourse performance (see Vilà-Giménez and Prieto, 2021, for a review). On the one hand, longitudinal studies have documented that early non-referential (beat) gestures (produced in parent–child naturalistic interactions) predict better structured narrative productions in later stages of children’s language development (Vilà-Giménez et al., 2021a). On the other hand, there is also evidence that both asking children to observe and encouraging them to produce non-referential (beat) gestures in a brief narrative training task also boosted children’s narrative performance in terms of narrative structure and fluency scores (Vilà-Giménez et al., 2019; Vilà-Giménez and Prieto, 2020). From this evidence, showing that non-referential gestures help boost not only children’s cognitive processes (i.e., information recall and narrative comprehension) but also narrative performance, we hypothesize that these gestures have a key multimodal framing function in narrative speech (i.e., the function to update common ground through marking IS that facilitates information processing for the listener, structuring the discourse) ultimately bootstrapping effective communication between speaker and listener. Further, as it has already been demonstrated that gestures associate with prosodic prominence, which in and of itself may serve as a multimodal cue to IS, it is important to assess to which point non-referential gestures play a role in IS-marking. It may be a child’s ability to recognize new information and mark it multimodally through gesture that allows for better discourse planning and construction, ultimately leading to these gains in narrative production.

While a good amount of research has been devoted to analyzing the development of IS-marking through morphosyntactic means in different types of children’s discourse, there is scarce evidence about how children mark IS gesturally, and how these changes over time. All in all, there is a clear need to analyze how children use both referential and non-referential gestures to mark IS to better assess the discourse-pragmatic functions of these different gesture dimensions and their relevance in children’s multimodal narrative speech.

### Research Questions and Hypotheses

The current study will analyze from a longitudinal perspective how children use referential and non-referential gestures to mark IS in narrative discourse at two different stages of language development, namely at 5–6 years of age, and again at 7–9 years of age. The “Audiovisual corpus of Catalan children’s narrative discourse development” (Vilà-Giménez et al., 2021b) was taken as a point of departure because this narrative corpus includes a set of 332 narratives produced by 83 children at these two points of development. IS-marking was analyzed by including three complementary dimensions, namely Topic/Comment, Focus/Background, and Referent Status.

Two main research questions will be addressed: (1) Do referential and non-referential gestures differ in how they co-occur with IS in children’s narrative discourse? and (2) How does the gestural marking of IS develop over time? Considering the first research question, we hypothesize that non-referential gestures will have a stronger tendency to mark topics, focus information, and newer referents, while referential gestures will mark accessible referents, provided the theoretical conceptions and empirical evidence about gesture dimensions and IS (see Section “Relationship Between Gesture and Information Structure” above), and the effects of non-referential gestures in framing children’s narrative discourse performance (see Section “Motivation of the Study: The Role of Non-referential Gestures in Children’s Discourse Development” above). Second, following up on previous research suggesting that the development of morphosyntactic IS-marking in the school-age period (i.e., at 7–9 years of age) develops as children’s discursive and narrative abilities evolve over time (e.g., Peterson and Dodsworth, 1991; Vion and Colas, 1998; Küntay, 2002), we predict that we will be able to already observe gestural patterns of IS-marking by the age of 5–6, but a clearer use will appear at the age of 7–9, which is the developmental time frame in which research has shown that children master reference introduction. Moreover, we also predict that by the age of 7, children will be able to successfully use non-referential gestures to mark IS, i.e., focused information and new referents, as it is when there is a significant increase in non-referential gesture use (e.g., Graziano, 2009; Florit-Pons et al., 2020).

## Materials and Methods

### Corpus

In order to assess the link between IS-marking and gestures over time, a longitudinal design was employed using the ‘‘Audiovisual corpus of Catalan children’s narrative discourse development’’^2^. The corpus consists of a total of 83 children (43 girls and 40 boys) carrying out a narrative retelling task at two time points in development, namely at the ages of 5–6 (*M* = 5.9, *SD* = 0.55; henceforth, Time 1), and two years later, at 7 to 9 years of age (*M* = 7.98, *SD* = 0.60; henceforth, Time 2).

At both time points, the children were asked to watch two wordless cartoons and retell the events of the cartoon to the experimenter, who was not familiar to the participants (i.e., was not a regular figure that they encountered at home or at school), and most did not remember her (the experimenter) at Time 2. The retelling was presented in the form of a game, such that the experimenter who pretended not to know the plot of narratives, had to guess which story the child had retold based on a set of images. These cartoons were approximately 41--50 *s* in length and were about a small mouse and his elephant friend who encountered some sort of difficulty and had to find a solution (*Die Sendung mit der Maus*; Westdeutscher Rundfunk Köln^3^). Each participant was randomly assigned two stories (out of four) to retell at Time 1 and the same two stories at Time 2. Each time, the first story they had to retell had only one character, the mouse, and the second story had two characters, the mouse and the elephant. The children told the same stories to the same experimenter at both times. For further details on the procedure, please refer to Vilà-Giménez et al. (2021b).

The corpus contains a total of 332 narratives (83 children × 2 stories × 2 time points). However, five children were excluded from the analysis as they did not perform a single gesture either at Time 1 or at Time 2. Overall, the current analysis is based on the narratives from 78 participants, which totaled approximately 141 min of multimodal narrative discourse.

### Data Coding

Each narrative was first imported into ELAN (Sloetjes, 2017), where it was transcribed orthographically. While the entire corpus was annotated for a number of bodily features, this goes beyond the scope of the current study, and thus the rest of the description will be in reference to the annotation of manual gestures. The subsections below describe how manual gesture and IS were coded, and how the relationship between the two was analyzed.

#### Gesture Coding

As previously described, we hold that gestures can be largely divided into two categories. Here, we use the term referential gestures to refer to those gestures that have a clear referent in speech, identified by “pictorial” hand shapes or movements (i.e., iconicity and metaphoricity) or through deixis. We distinguish referential gestures from non-referential gestures, which do not visually portray speech content.

**FIGURE 5:**
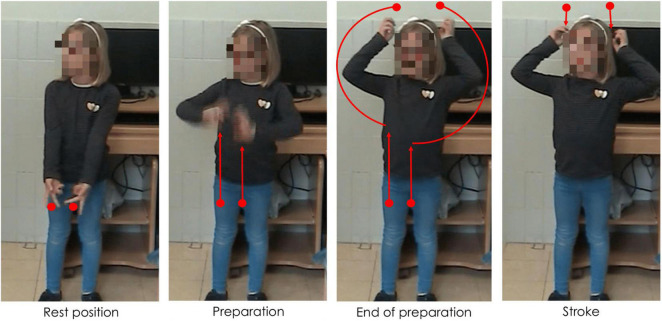
Caption of a referential iconic gesture while saying “la part de DALT” (“the UPPER part”). Capitalization indicates the word accompanied by a gesture.

**FIGURE 6:**
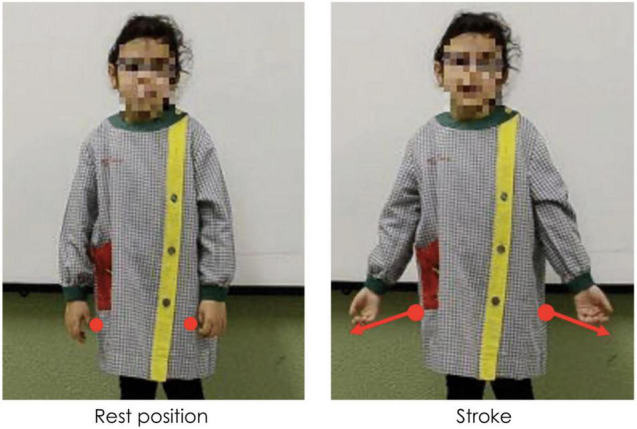
Caption of a biphasic non-referential gesture while saying “hi havia un COTXET” (“there was a SMALL CAR”). Capitalization indicates the word accompanied by a gesture.

The annotation of manual gestures was conducted in two steps. First, an initial pass without sound was carried out to identify gesture phasing, and, particularly, the strokes of gestures roughly identified by the form of the hands as either having clearly referential (i.e., iconic, metaphoric, or deictic) forms or through other aspects such as the trajectory shape, direction, and speed. A second pass with audio allowed for a validation or rectification of the gesture phasing annotation, and based on the content of speech, gesture referentiality was determined. For the purposes of the current study, each gesture stroke was classified as being *referential* (iconic, metaphoric, or deictic) or *non-referential* in nature. While non-referential gestures can have different hand shapes and complex movement phases (Shattuck-Hufnagel et al., 2016; Prieto et al., 2018; Shattuck-Hufnagel and Prieto, 2019), most of the non-referential gestures encountered in the database were small monophasic or biphasic movements, which could occur in a number of directions (e.g., bending the wrist up, or a slight movement of the hand down). This style of gesture is not incoherent with what other studies have termed “proto-beats” (e.g., Koutalidis et al., 2020). Figure 5 shows an example of a referential gesture while Figure 6 shows an example of a non-referential gesture produced by two participating children. Conventional (or emblem) gestures were also coded but were excluded from analysis (*N* = 28) as they have been described as conventional signs with systematic rules about form and are culturally defined (McNeill, 1992).

Once gesture strokes and their referentiality properties were annotated, for each gesture stroke, a “target word” was identified from the orthographic transcription. The target word refers to the lexical item or items in speech that temporally co-occurred with the gesture stroke. The target word tier thus served as the basis for matching the gesture strokes with the different levels of IS.

#### Information Structure Coding

The simplified version of the LISA labeling guidelines described in Götze et al. (2007) and Ritz et al. (2008) was used. This annotation scheme is performed on three levels which are non-mutually exclusive: the annotation of (a) information that may or may not move the discourse forward (referred to simply as *Focus* as opposed to *Background*), (b) sentence constituents referring to thematic material or predication (*Topic* vs. *Comment*), and (c) the information status of discourse referents in speech (*Referent Status*) (see Section “Information Structure” for a detailed description of the IS levels). Following LISA, the most precise IS level was annotated first (i.e., Referent Status). For each target word, labelers first identified if the target word was a discourse referent in speech. If so, the referent’s status was then labeled as being new, given, or accessible. After annotating the Referent Status, the labelers had to determine whether any annotated referent was an aboutness-topic (i.e., a noun phrase which receives the predication), following an aboutness-topic test (Götze et al., 2007, p. 165). Parts of the utterance that “specify the time or the location at which the event/state denoted by the rest of the clause takes place/holds” were identified as frame-setting topics (most often temporal or locative prepositional phrases, adverbial phrases, and subordinate clauses indicating spatial or temporal locations) (Götze et al., 2007, p. 167). Stretches of speech that were not identified as topics were then annotated as comment constituents. Finally, the broadest level of IS is annotated (i.e., Focus/Background), determining which parts of the utterance are moving the discourse forward (subsequently annotated as being within a focus constituent). Portions of speech that were not deemed to be serving to develop discourse were then annotated as background.

#### Coding Reliability

Both percent agreement and Gwet’s Agreement Coefficient 1 (AC1) (Gwet, 2008) were used to assess the coder reliability. Gwet’s AC1 was chosen to assess reliability as it is resistant to the “Kappa Paradox,” a situation where one category is observed significantly more than another, which in turn affects the marginal totals in the calculation of chance agreement, effectively reducing the kappa statistic regardless of high agreement (e.g., Cicchetti and Feinstein, 1990; Feinstein and Cicchetti, 1990; Krippendorff, 2004). Gwet’s AC1 uses the same formula as the Kappa (and thus can be interpreted in a similar way) but calculates the agreement from chance to correct for these biases. For further explanation of Gwet’s AC1, see Dettori and Norvell (2020).

Reliability analysis was conducted using the *irrCAC* package in R (Gwet, 2019). It was assessed using 64 narratives (which represents approximately 20% of the entire database and included 147 gestures), where a second coder assigned values for each gesture in terms of its referentiality and IS levels (Background/Focus, Topic/Comment, and Referent Status in terms of new, accessible, or given referents). Agreement was good for both gesture referentiality [Percent agreement: 78.2%; AC1 = 0.731 (95% CI, 0.648 to 0.815), *p* < 0.001] and Referent Status [Percent agreement: 86.8%; AC1 = 0.802 (95% CI, 0.661 to 0.943), *p* < 0.001]. Agreement was moderate for both Focus/Background [Percent agreement: 75.2%; AC1 = 0.706 (95% CI, 0.686 to 0.785), *p* < 0.001] and Topic/Comment [Percent agreement: 67.2%; AC1 = 0.611 (95% CI, 0.591 to 0.632), *p* < 0.001].

Our reliability analyses showed some disagreements among the coders, which might have been caused by ambiguous cases that are difficult to classify. For instance, some narratives contained repetitive actions (e.g., a mouse hangs socks to dry, and the wind blows the socks off the clothesline, an action that is repeated 3 times). The subsequent repetitions of the socks getting blown off the line can be interpreted as background in that it does not advance the plotline much, as the same action is recurring. Alternatively, the repetition of each action can be seen as an individual updating element of the plotline, ultimately moving the discourse forward. Similarly, for discrepancies in Topic/Comment, we have observed ambiguous cases in the annotation of prepositional phrases (e.g., “l’elefant *amb la trompa* toca l’arbre,” English translation: “the elephant *with the trunk* touched the tree,” ambiguous prepositional phrase in italics) which can be interpreted as an aboutness topic (who touched the tree? – The elephant with the trunk), a frame-setting topic (which elephant touched the tree? – the one with the trunk), or as a comment (What about the elephant? – He touched the tree with his trunk).

### Statistical Analyses

Because Focus/Background, Topic/Comment, and Referent Status are non-mutually exclusive levels of IS, three separate Linear Mixed Effects Models (LME) were run, one for each level. Before running the LME models, the data was prepared so that gestures that occurred without speech or with words that do not contribute to the IS of the narrative (e.g., “and” as a conjunction between informative clauses) were excluded from the analysis. Further, a total of 10 referential gestures (3 from Time 1 and 7 from Time 2) were excluded from analyses because the stroke did not associate with a single word or constituent but rather lasted during entire clauses and covered multiple IS categories.

The three LME models were run using the *lme4* package in R (Bates et al., 2015) to analyze the link between gesture referentiality (referential vs. non-referential gestures) and IS (i.e., in terms of Focus/Background, Topic/Comment, and Referent Status). The dependent variable in all three models was the number of gestures produced. To determine the random effects structure for each model, a series of LME models were run using all the potential combinations of random effects variables, from the most complex structure to a basic model containing no random effects. Models that did not produce any convergence issues were then compared using the “compare performance” function from the *performance* package (Lüdecke et al., 2021) to select the best-fitting model for the data.

In the first model, the fixed factors were Time (2 levels: Time 1 and Time 2), Gesture Referentiality (2 levels: Referential and Non-referential), and Focus/Background (2 levels: Focus and Background), along with their 3-way interaction. The random-effects structure included by-Participant varying intercepts and by-Participant varying slopes for the interaction between Time and Gesture Referentiality. In the second model, Time (2 levels: Time 1 and Time 2), Gesture Referentiality (2 levels: Referential and Non-referential), and Topic/Comment (3 levels: Aboutness Topic, Frame-Setting Topic, and Comment) were set as fixed factors, along with their 3-way interaction. The random-effects structure included by-Participant varying intercepts and by-Participant varying slopes for Time and Gesture Referentiality. In the third model, Time (2 levels: Time 1 and Time 2), Gesture Referentiality (2 levels: Referential and Non-referential), and Referent Status (3 levels: New, Given, and Accessible) were set as fixed factors, along with their 3-way interaction. In addition to the exclusion criteria previously mentioned, gestures that did not associate with a noun phrase or prepositional phrase (i.e., a discourse referent) were excluded. Finally, two participants were discarded from the LME for Referent Status because they produced no gestures at either Time 1 or Time 2 that aligned with a discourse referent. The random-effects structure included by-Participant varying intercepts and by-Participant varying slopes for Time and Gesture Referentiality. Omnibus test results are described below, along with the results from a series of Bonferroni pairwise tests carried out with the *emmeans* package (Lenth, 2021), which includes a measure of effect size (*via* Cohen’s *d*).

## Results

To characterize the data in a more comprehensive way and highlight the variability among children and differences between the two time points, the following paragraph describes the descriptive statistics for the narrative productions. In terms of speech, the average length of the children’s narrations at Time 1 was 27.07 *s* (*SD* = 10.45) with an MLU of 7.39 words and 28.37 *s* (*SD* = 9.49) with an MLU of 7.05 words at Time 2. In terms of gesture, 868 gestures were annotated (173 referential and 108 non-referential gestures at Time 1; 277 referential and 310 non-referential gestures at Time 2). Gesture rate was calculated both in terms of the average number of gestures per utterance and gestures per narration. At Time 1, children performed an average of 0.097 gestures per utterance (or an average of 3.12 gestures per narration), while at Time 2, children performed an average of 0.229 per utterance (or an average of 4.93 gestures per narration).

The three statistical models showed a significant main effect of Time, indicating that there were more gestures at Time 2 than at Time 1, and a significant main effect of Gesture Referentiality, suggesting that there were more non-referential gestures than referential gestures. Table 1 shows the main effects and *post hoc* results for each model.

**TABLE 1:** Main effect and *post hoc* results for the three statistical Linear Mixed Effects Models (LME) models.

	Model 1 for Focus/Background	Model 2 for Topic/Comment	Model 3 for Referent Status
Main effect of Time	χ^2^(1) = 19.641, *p* < 0.001	χ^2^(1) = 20.729, *p* < 0.001	χ (1) = 11.188, *p* < 0.001
*Post hoc* results for Time	*t*(92.7) = −5.209, *SE* = 0.144, *p* < 0.001	*t*(92.1) = −5.361, *SE* = 0.0923, *p* < 0.001	*t*(92.7) = −5.209, *SE* = 0.144, *p* < 0.001
Main effect of Gesture Referentiality	χ^2^(1) = 15.351, *p* < 0.001	χ^2^(1) = 17.348, *p* < 0.001	χ^2^(1) = 17.011, *p* < 0.001
*Post hoc* results for Gesture Referentiality	*t*(77) = 3.918, *SE* = 0.0129, *p* < 0.001	*t*(77) = 4.165, *SE* = 0.0813, *p* < 0.001	*t*(77) = 3.918, *SE* = 0.0129, *p* < 0.001

The first LME model considering Focus/Background reported a main effect of Focus/Background [χ^2^(1) = 163.461, *p* < 0.001], showing that there were significantly more gestures marking focus constituents than background constituents [*t*(697) = −13.206, *SE* = 0.129, *p* < 0.001]. The 3-way interaction was also found to be significant [χ^2^(1) = 9.665, *p* = 0.002].

**FIGURE 7:**
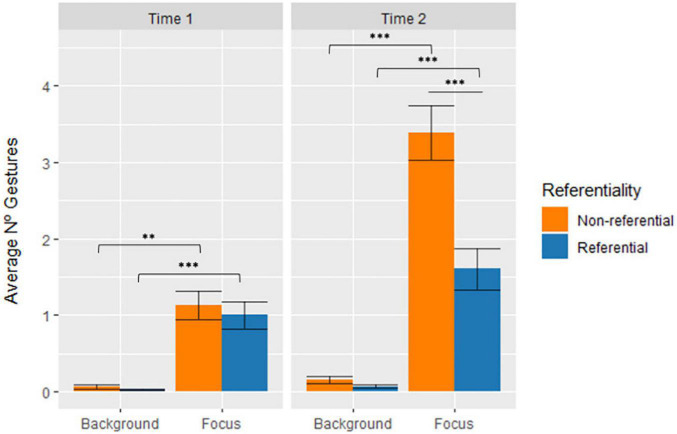
Average number of gestures per child across Time, Gesture Referentiality, and Focus/Background. Error bars represent standard error. * stands for *p* ≤ 0.05; ^**^ stands for *p* ≤ 0.01; and ^***^ stands for *p* ≤ 0.001.

Taking into account that the interaction can be interpreted from three different perspectives, if we compare the two categories, we observe that at Time 1 non-referential gestures marked significantly more focus than background constituents (*d* = −0.572, *p* = 0.004), a tendency which was also found for referential gestures, which marked significantly more focus constituents than background (*d* = −0.524, *p* < 0.001). The same patterns of association were found at Time 2, both for non-referential (*d* = −1.738, *p* < 0.001) and referential gestures (*d* = −0.524, *p* < 0.001). Comparing across Time, the interaction shows that non-referential gestures at Time 2 marked more focus constituents than at Time 1 (*d* = −121.40, *p* < 0.001). Referential gestures, however, showed no significant differences in the marking of Referent Status from Time 1 to Time 2. Finally, by comparing gesture dimensions, the interaction indicates that it is only at Time 2 that there are significantly more non-referential gestures marking focus constituents than referential gestures (*d* = 0.955, *p* < 0.001), while no differences between these two gesture dimensions were observed at Time 1. Figure 7 shows the average number of referential and non-referential gestures per child associated with Focus/Background in the two time points in development (significant differences are marked with an asterisk).

The second LME model for Topic/Comment (i.e., aboutness topic, frame-setting topic or comment), showed a significant main effect of Topic/Comment [χ^2^(2) = 258.529, *p* < 0.001], indicating that there were more gestures associated with comment than with aboutness topic [*t*(1161) = 14.091, *SE* = 0.0993, *p* < 0.001] or frame-setting topic [*t*(1161) = 14.075, *SE* = 0.0993, *p* < 0.001], with no significant difference between the marking of the two types of topic. The 3-way interaction between Gesture Referentiality, Time, and Topic/Comment was found to be significant [χ^2^(2) = 11.45, *p* = 0.003].

**FIGURE 8:**
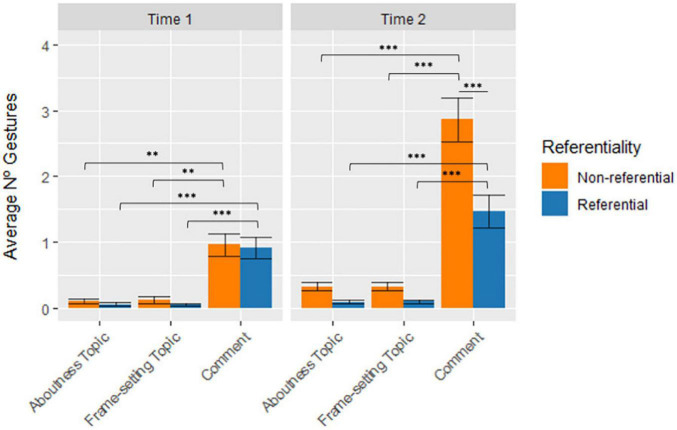
Average number of gestures per child across Time, Gesture Referentiality, and Topic/Comment. Error bars represent standard error. * stands for *p* ≤ 0.05; ^**^ stands for *p* ≤ 0.01; and ^***^ stands for *p* ≤ 0.001.

From the perspective of Topic vs. Comment, the interaction revealed that at Time 1, non-referential gestures marked comment constituents significantly more than either aboutness (*d* = 0.591, *p* = 0.006) or frame-setting (*d* = 0.582, *p* = 0.007) topics. The same relationship was found for referential gestures (aboutness topics: *d* = 0.591, *p* < 0.001; frame-setting topics: *d* = 0.599, *p* < 0.001). Further, the same patterns of association were found at Time 2, where non-referential gestures marked comment constituents significantly more than aboutness (*d* = 1.764, *p* < 0.001) or frame-setting (*d* = 1.764, *p* < 0.001) topics, and with referential gestures following suit (aboutness topics: *d* = 0.963, *p* < 0.001; frame-setting topics: *d* = 0.958, *p* < 0.001). Comparing across Time, we see that non-referential gestures at Time 2 marked significantly more comment constituents (*d* = −1.325, *p* < 0.001) than at Time 1. Referential gestures, however, showed no significant differences in the marking of topic or comment constituents from Time 1 to Time 2. Finally, by comparing gesture dimensions, the interaction indicates that it is only at Time 2 that there are significantly more non-referential gestures marking comment constituents more than referential gestures (*d* = 0.967, *p* < 0.001). That is, both gesture dimensions at both time periods are associated with comment constituents significantly more than topics. Figure 8 shows the average number of referential and non-referential gestures per child associated with Topic/Comment in the two time points in development (significant differences are marked with an asterisk).

The last LME analysis for Referent Status showed a significant main effect of Referent Status [χ^2^(2) = 74.101, *p* < 0.001], which reveals that the number of gestures associated with new referents in speech was significantly higher than the gestures associated with given referents [*t*(1056) = −7.882, *SE* = 0.051, *p* < 0.001] or accessible referents [*t*(1056) = −10.097, *SE* = 0.051, *p* < 0.001]. A significant 3-way interaction between Time, Gesture Referentiality, and Referent Status was also reported [χ^2^(2) = 21.071, *p* < 0.001].

**FIGURE 9:**
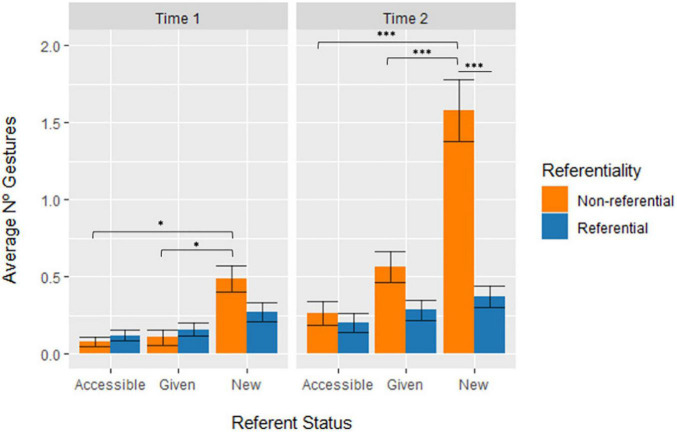
Average number of gestures per child across Time, Gesture Referentiality, and Referent Status. Error bars represent standard error. * stands for *p* ≤ 0.05; ^**^ stands for *p* ≤ 0.01; and ^***^ stands for *p* ≤ 0.001.

The interaction can be interpreted from three perspectives: by comparing the marking of Referent Status, by comparing the difference between Time 1 and Time 2, and by comparing the gesture dimensions. From the perspective of the marking of Referent Status, the interaction showed that at Time 1, non-referential gestures marked significantly newer than given (*d* = −0.531, *p* = 0.026) or accessible referents (*d* = −0.567, *p* = 0.012). However, referential gestures showed no significant differences between the marking of Referent Status at Time 1. The same tendency is found at Time 2, where non-referential gestures marked significantly newer than given (*d* = −1.409, *p* < 0.001) or accessible referents (*d* = −1.83, *p* < 0.001), while referential gestures still showed no significant differences between the marking of Referent Status. Comparing across Time, we see that non-referential gestures at Time 2 marked more significantly given (*d* = −0.641, *p* = 0.021) and new referents (*d* = −0.137, *p* < 0.001) than at Time 1. Referential gestures, however, showed no significant differences in the marking of Referent Status from Time 1 to Time 2. Finally, by comparing gesture dimensions, we also see that it is only at Time 2 that there are significantly more non-referential gestures marking new referents than referential gestures (*d* = 1.684, *p* < 0.001). Figure 9 shows the average number of referential and non-referential gestures per child associated with Referent Status in the two time points in development (significant differences are marked with an asterisk).

## Discussion and Conclusion

The current study describes the results of a longitudinal investigation of children’s development of multimodal IS-marking by focusing on the analysis of children’s narrative speech at two time points in development (i.e., at the ages of 5–6, and two years later). The two main objectives of the study were the following: (a) to analyze how referential and non-referential gestures are employed in children’s narrative discourse to mark IS (in terms of Focus/Background, Topic/Comment, and Referent Status), and (b) to assess the development of these IS-marking functions at two different time points. To our knowledge, the present study is the first to longitudinally investigate how referential and non-referential gestures mark IS considering three different dimensions.

First, concerning the role of gesture in marking Focus/Background, the results revealed that both referential and non-referential gestures associate significantly more with focused information than with non-focused (i.e., background) information in children’s narrative discourse, and that this relationship is already established at 5 years of age. This finding corroborates and expands on existing research on multiple fronts. On the one hand, in the field of gesture studies, our findings provide empirical support for previous claims that gestures (regardless of gesture referentiality) are more likely to co-occur with less accessible information (i.e., newer information that propels the discourse forward; McNeill, 1992, 2005). In fact, we found that it is not only non-referential gestures which are typically related to focus-marking, but referential gestures as well, reflecting the results of previous studies that have shown that gestures tend to co-occur with “new information,” whether that be in terms of rhematic elements or discourse-new referents (e.g., Marslen-Wilson et al., 1982; McNeill, 1992; Levy and Fowler, 2000; Gullberg, 2003; Yoshioka, 2008; Debreslioska et al., 2013; Debreslioska and Gullberg, 2019). This finding is not entirely surprising, as according to McNeill’s (1992) Phonological Synchrony Rule, gestures tend to associate with pitch accentuation (see also Yasinnik et al., 2004; Jannedy and Mendoza-Denton, 2005; Shattuck-Hufnagel and Ren, 2018; among many others), and in the domain of prosodic studies, it is commonly held that pitch accentuation marks focus. Therefore, if gestures generally associate with pitch accents, which in turn mark focus, it is only natural that gestures (regardless of referentiality) have a tendency to mark focused constituents. This has also been already shown in the gesture literature (e.g., see Section “Relationship Between Gesture and Information Structure”).

Second, with regards to the multimodal distinction between topic and comment, we found that both gesture dimensions are overwhelmingly produced during comment constituents, while very few gestures mark topics at both points in narrative development. Even though not significant, we found a tendency for non-referential gestures to mark topics more than referential gestures at Time 2. This tendency can be read taking into account McNeill’s theory of Communicative Dynamism and gesture production: non-referential gestures are more likely to go with thematic information (i.e., topics, see Figures 2, 3) with lower communicative dynamism than referential gestures.

Third, regarding the gestural marking of Referent Status, our results showed that children begin using non-referential gestures to mark new referents significantly more than given or accessible referents by 5–6 years of age (i.e., at Time 1). By contrast, referential gestures in our data did not significantly mark novelty effects on referents. Crucially, this suggests that at this point in development, non-referential gestures may have an important role in the organization of discourse (particularly for the introduction of novel discourse entities) which is not shared by referential gestures. Further, the use of non-referential gestures is already in place by 5 years of age and increases significantly by 7 years of age, coinciding with the time frame when children’s use of non-referential gestures increases (e.g., Florit-Pons et al., 2020). The findings about the use of non-referential gestures to mark new information in children’s speech empirically confirm some recent claims on the pivotal discourse-pragmatic role of non-referential gestures (McNeill, 1992; Colletta et al., 2015; Shattuck-Hufnagel et al., 2016). On the one hand, such structural properties of non-referential gestures—such as introducing new referents as in the present study—adds evidence to the beneficial effects obtained when training children with observing (Vilà-Giménez et al., 2019) and encouraging them (Vilà-Giménez and Prieto, 2020) to produce these gestures. On the other hand, our results also reinforce the predictive value of non-referential gestures found in Vilà-Giménez et al.’s (2021a) study, supporting the idea that these gestures may play an important discourse-pragmatic role starting early in children’s language development. Overall, our results strongly support the view that the bootstrapping and predictive role of non-referential gestures in children’s narrative development has its roots in their pragmatic and discursive properties, and they may be key when explaining why non-referential gestures have been reported to be so relevant in children’s narrative discourse abilities (see Vilà-Giménez and Prieto, 2021, for a review).

The results shown in Figure 9 further revealed two interesting patterns. On the one hand, about a quarter of the gestures (23.8% at Time 1; 27.5% at Time 2), both referential and non-referential, were also associated with given referents. This result seems to contrast with previous studies suggesting that gestures associate with discourse-new referents (e.g., Marslen-Wilson et al., 1982; Levy and Fowler, 2000; Gullberg, 2003; Yoshioka, 2008; Debreslioska et al., 2013; Debreslioska and Gullberg, 2019). However, given referents can be considered of two types based on their accessibility. “Reintroduced” referents occur when a certain distance has passed since their last mention, or they contrast with another referent and are thus reintroduced using the full noun phrase form. This is distinguished from “maintained” referents which are highly accessible and often produced in speech with zero anaphora or a pronoun. Interestingly, Debreslioska et al. (2013) indeed found that referential (iconic) gestures co-occurred with given referents and the viewpoint of the gestures (character-viewpoint vs. observer-viewpoint) was in fact modulated based on the accessibility of the given referent. Upon a brief inspection of our own data, we found that gestures that co-occurred with given referents were almost entirely produced with reintroduced referents in their full noun phrase form. However, future studies should include a more refined coding of IS-marking that includes further categorizations for referent accessibility to better understand how gesture production is modulated.

On the other hand, accessible referents were the least often marked gesturally. This finding also seems to contrast with studies in adults (e.g., Debreslioska and Gullberg, 2020b; Im and Baumann, 2020). However, this may be related to the fact that these referents are already active in the children’s mind and may be the most difficult category to assess their place in perspective-taking. In other words, as a given referent has been explicitly said, the child can easily deduce that the listener is aware of the referent’s existence. Comparatively, if a discourse referent is brand new, the child can deduce rather easily that the listener will benefit from additional multimodal cues to interpret it as such. Accessible referents, however, may already be considered more “given” from the children’s perspective, which may not align with the listener’s perspective of it being a less-retrievable referent. The finding that adults mark more accessible referents may thus reflect a better flexibility in perspective-taking than children (e.g., Jacques and Zelazo, 2005). See below for further discussion about the current findings in relation to the development of more complex cognitive processes.

From a developmental perspective, the results of the current study help expand our knowledge on the development of children’s multimodal narrative development, specifically of IS-marking. Interestingly, the data presented in this article has provided empirical evidence that at Time 2 (i.e., at 7–9 years of age) non-referential gestures are used more than referential gestures to mark comment and focus constituents, along with new referents in narrative discourse. This coincides with the time frame in which children hit a spurt in the production of non-referential gestures, use them in more complex discourses such as narratives (see e.g., Florit-Pons et al., 2020), and make use of non-referential gestures for other pragmatic functions, such as discourse cohesion (e.g., Colletta et al., 2010, 2015; Shattuck-Hufnagel et al., 2016). Moreover, our data has shown that even though children used gestures regardless of referentiality to mark new information/predication at all levels of IS (i.e., focus constituents, comment, and new referents) more than old/shared or thematic information (i.e., background constituents, topic, and given and accessible referents), it is specifically at Time 2 that non-referential gestures are being used significantly more often than referential gestures to signal new information. Our findings help expand our knowledge about the relationship between gesture and IS by adding a distinction between referential and non-referential gestures, as previous studies claiming that gestures introduced new referents focused on referential gestures, or did not distinguish gesture referentiality (e.g., Marslen-Wilson et al., 1982; Levy and Fowler, 2000; Gullberg, 2003; Yoshioka, 2008; Debreslioska et al., 2013; Debreslioska and Gullberg, 2019). Our results demonstrate that as children reach 7–9 years of age, non-referential gestures become a key player in the marking of novel information.

The use of manual non-referential gestures seems to represent an important intermediate step in the development of IS-marking. Indeed, a study by Esteve-Gibert et al. (2021) suggests that French children at the age of 4–5 years initially mark contrastive focus with head nods. Then, by 7–9 years of age, children are capable to start using morphosyntactic devices to reference discourse entities in an adult-like manner, specifically in introducing or marking new referents (e.g., Hickmann, 1980, 1982; Bamberg, 1986; Kail and Hickmann, 1992; Vion and Colas, 1998; Küntay, 2002). Finally, around age 12, children are able to use abstract pointing gestures, where referents are established in a mental space and are subsequently pointed to (McNeill, 1992, p. 60). Thus, our findings seem to suggest that the ability to mark IS in speech and gesture develops in parallel fashion, where potentially changes in the pragmatic complexity of gesture (i.e., the non-referential “burst” at this developmental stage) occur just before changes in speech. Furthermore, our findings are reinforced by evidence across all three independent dimensions of IS, suggesting that non-referential gestures serve to mark elements that update common ground.

Importantly, we suspect that the ability to use manual non-referential gestures to mark IS might be related to children’s more complex cognitive and linguistic processes, such as narrative or pragmatic abilities (see Vilà-Giménez and Prieto, 2021, for a review), or more complex cognitive skills, such as Theory of Mind (ToM; e.g., Carmiol and Sparks, 2014; Graf and Davies, 2014), which emerge and evolve at this stage in development. While research has demonstrated the link between non-referential gestures and narrative development, as far as we know, there is no previous study that assesses how these gestures relate to ToM. Interestingly, ToM abilities are key in narrative development, as having the capacity to understand and attribute mental states to oneself and to others can help better understand the story and the character’s perspectives, and at the same time to better retell the story (e.g., Gamannossi and Pinto, 2014). We believe that the ability to use gesture for IS marking is tightly linked to children’s general communication abilities, enhancing the properties of gestures to structure discourse to improve communication. There is thus a clear need for further investigations about the timeline of acquisition of IS through several grammatical and multimodal means, and how it relates with the development of other cognitive abilities, such as ToM.

With respect to our assessment of the adequacy of Götze et al.’s (2007) IS annotation scheme for children’s narrative discourse, our assessment is positive. Most theories of IS have been largely based on fictitious speech samples and Question/Answer pairs, and limited studies have applied such theories to natural discourse. The annotation system employed in the present study offers a text-based annotation, which limits bias from prosodic cues to IS, as well as a clearly defined annotation procedure, which allows for fairly quick and straightforward data annotation across well-established dimensions of IS. Some aspects of this system remain a bit vague and subjective (particularly in terms of identifying focus constituents which can lead to cases of ambiguity – see subsection “Coding Reliability” for some examples, and also Ritz et al., 2008, for issues regarding reliability), and it does not account for complex structures (such as nested foci, second occurrence focus, or primary and secondary foci; see e.g., Levy and McNeill, 1992; Riester and Baumann, 2013). Hence, other more complex annotation systems could be implemented [such as the Questions Under Discussion annotation guidelines by Riester et al. (2018), which focuses on the identification of focus constituents]. However, such systems are much more complex, time consuming, and go beyond the scope of the current study. In any case, we believe that by using such annotation systems to apply theories of IS to natural discourse, researchers may be able to refine some of the theoretical underpinnings of IS, such as disentangling notions of “focus” and “contrast.”

The current study presents some limitations in terms of annotation that prevent the results to be more directly compared to some previous investigations. Our study focuses only on referents that have been marked gesturally, while other studies (e.g., Debreslioska and Gullberg, 2020b; Im and Baumann, 2020) consider all discourse referents, analyzing the frequency with which referents were marked by gestures. Despite that, we believe that the previously reported findings are complementary with ours. In the present study, we offer the perspective of *what gestures are actually marking in speech* rather than the frequency with which different referent types are marked gesturally, and therefore we think it would be interesting for further analyses to consider assessing the relationship between gesture and Referent Status combining both approaches. Further, this study did not take into account interactions between levels of IS. While future studies should indeed look into these interactions (responding to more specific research questions such as “Do gestures have a tendency to mark given referents more when they are within contrastive focus constituents?”), the current study limits the research questions to looking at each level of IS individually as the main goal is to assess longitudinal development. By looking at the levels independently, we are still able to assess that non-referential gestures are differentially marking elements of discourse that aim to update the common ground between the speaker and the listener, while referential gestures do not share this same pattern in Catalan narratives by children.

Our investigation has left some open questions for future work. First, it would indeed be interesting for further studies to include comprehensive prosodic annotations to better understand how gesture and prosody work together for the marking of IS (while keeping in mind the limitations of IS annotation independent of prosody as described in the preceding paragraph). Second, while our results help us understand the pragmatic IS-marking properties of non-referential gestures, less is known about how the use of gestures combines with other morphosyntactic strategies, such as in topicalization, zero anaphora (e.g., “Mark had a shower and Ø brushed his teeth”) and cleft sentences. For instance, existing research has shown that for focus-marking, non-referential (beat) gestures associate with prosodic markers of focus, while metaphoric gestures tend to associate with syntactic markers of focus (Ferré, 2014). In terms of Referent Status, when reintroducing a given referent using pronouns or zero anaphora, speakers do not use gestures at all (Debreslioska et al., 2013). Therefore, it would be interesting to see whether similar patterns are found in children’s narrative discourse as well. Third, further research would be needed to examine whether children’s use of non-referential gestures produced in earlier stages of development (e.g., at the age of 3–4 when IS-marking is just starting to be acquired) can predict their ability to reference discourse entities.

To our knowledge, this is the first study to find that non-referential gestures do function pragmatically as markers of elements that update common ground between listener and speaker by investigating three independent levels of IS. However, we believe that this is merely one potential function that non-referential gestures may have. Indeed, these gestures can have other functions, such as stance marking, discourse structure marking, and emphasizing (e.g., McNeill, 1992; Loehr, 2012; Rohrer et al., 2020), and even be polyfunctional in nature (i.e., having multiple pragmatic functions simultaneously, a point held by multiple authors including McNeill, 2000; Kendon, 2017; Lopez-Ozieblo, 2020). Our study has elucidated one such pragmatic function of these gestures and future studies should investigate other potential pragmatic functions or their interactions.

In conclusion, this study helps refine our knowledge about the multimodal development of gesture and IS in children’s narrative discourse. Our research has shown the key discourse-pragmatic value that non-referential gestures serve in children’s more complex discourses, such as narratives, specifically in the marking of elements that update the common ground between listeners and speakers, such as focus constituents, predication, and discourse-new referents. These findings help explain why these gestures can act as visual highlighters of discourse structure and help bootstrap children’s oral narrative discourse performance. Overall, in the upcoming years, developmental research will need to gain a more refined knowledge of how children’s gestural and discursive abilities, specifically IS-marking abilities, go hand in hand and develop over time.

## Data Availability Statement

The raw data supporting the conclusions of this article will be made available by the authors, without undue reservation.

## Ethics Statement

Ethical review and approval was not required for the study on human participants in accordance with the local legislation and institutional requirements. Written informed consent to participate in this study was provided by the participants’ legal guardian/next of kin. Written informed consent was obtained from the minor(s)’ legal guardian/next of kin for the publication of any potentially identifiable images or data included in this article.

## Author Contributions

PLR, JF-P, IV-G, and PP contributed equally to the development of the research questions, the experimental design, and the discussion of the results. IV-G collected the data for the Audiovisual Corpus of Catalan Children’s Narrative Discourse Development. JF-P carried out most of the data annotation of the corpus, with the aid of IV-G and two other annotators. The analysis was mainly carried out by PLR with the aid of JF-P. JF-P and PLR were jointly in charge of writing the article, with substantive feedback from IV-G and PP. All authors contributed to the article and approved the submitted version.

## Conflict of Interest

The authors declare that the research was conducted in the absence of any commercial or financial relationships that could be construed as a potential conflict of interest.

## Publisher’s Note

All claims expressed in this article are solely those of the authors and do not necessarily represent those of their affiliated organizations, or those of the publisher, the editors and the reviewers. Any product that may be evaluated in this article, or claim that may be made by its manufacturer, is not guaranteed or endorsed by the publisher.

## Footnotes

1 IS-marking refers to the regular co-occurrence between certain aspects of speech (syntax/prosody/gesture) and certain types of IS constituents at different levels (i.e., Focus/Background, Topic/Comment, or New/Accessible/Given referents).

2 To see other projects and publications related to this corpus, please visit: https://osf.io/npz3w

3
www.wdrmaus.de
