# Supplementary material for: Children Use Non-referential Gestures in Narrative Speech to Mark Discourse Elements Which Update Common Ground
Source: Front Psychol. 2022 Jan 11;12:661339. doi: 10.3389/fpsyg.2021.661339 (PMC8787325; doi:10.3389/fpsyg.2021.661339)
Supplement: Supplementary file 1 [file Table_1.pdf]

Appendix A. Examples of the Information Structure Annotation extracted from the corpus[1]

Level	Examples	Translation to English
Focus / Background	[Hi havia un ratolí que estava passejant amb el seu elefant]$_{Foc}$	There was a mouse who was walking with his elephant.
	I després, [el ratolí i l'elefant trepitgen la petxina per obrir-la]$_{Foc}$	And then, the mouse and the elephant step on the seashell to open it.
	[El ratolí torna a estendre la roba]$_{Foc}$ i [el vent torna a bufar]$_{Foc}$, i llavors, [torna a estendre la roba]$_{Back}$ i [torna a bufar el vent]$_{Back}$	'The mouse hangs out the washing again and the wind blows again, and then, he hangs out the washing again and the wind blows again.'
	[Com que el balancí es movia]$_{Back}$, [amb el peu ha inflat l'arbre]$_{Foc}$	'Since the rocking chair was moving, with the foot he has inflated the tree.'
Topic / Comment	[Un ratolí de color taronja]$_{AB\text{-}Top}$ [estenia la roba]$_{Com}$	An orange mouse was hanging out the washing.
	[Al final]$_{FS\text{-}Top}$, [l'elefant i el ratolí]$_{AB\text{-}Top}$ [van tenir una idea]$_{Com}$	In the end, the elephant and the mouse had an idea
	[Un ratolí que portava un elefant amb un carretó]$_{AB\text{-}Top}$ [es troba un arbre de ferro]$_{Com}$	A mouse who is carrying an elephant in a cart finds a metal tree.
	I [cada vegada que ve el vent]$_{FS\text{-}Top}$ [li tira els mitjons a terra]$_{Com}$	And every time that the wind comes, blows the socks down.
	[El ratolí]$_{AB\text{-}Top}$, [després]$_{FS\text{-}Top}$, [fica els mitjons dins el fil d'estendre]$_{Com}$	The mouse then puts the socks inside the clothesline.

[1] Abbreviations: Foc = Focus; Back = Background; AB-Top = Aboutness Topic; FS-Top = Frame-setting Topic; Com = Comment; New = New referent; Given = Given referent; Acc = Accessible referent

Level	Examples	Translation to English
Referent Status	[El ratolí]$_{New}$ estava estenent [la roba]$_{Acc}$ i [el vent]$_{New}$ [la]$_{Given}$ tira [a terra]$_{Acc}$	‘The mouse was hanging out the washing and the wind blows it down.’
	[El ratolí i l’elefant]$_{New}$ estan caminant i es troben [una estructura feta de ferro]$_{New}$. [L’elefant]$_{Given}$ [la]$_{Given}$ toca i llavors [totes les peces de l’estructura]$_{Acc}$ cauen. Al final, [ells]$_{Given}$ construeixen [una muntanya russa]$_{Given}$.	‘The mouse and the elephant are walking and find a structure made of iron. The elephant touches it and then all the pieces of the structure start falling. Finally, they build a roller coaster.’
