# Supplementary material for: Children Use Non-referential Gestures in Narrative Speech to Mark Discourse Elements Which Update Common Ground
Source: Front Psychol. 2022 Jan 11;12:661339. doi: 10.3389/fpsyg.2021.661339 (PMC8787325; doi:10.3389/fpsyg.2021.661339)
Supplement: Supplementary file 2 [file Table_2.pdf]

Appendix B. Example of the coding. Sample narrative extracted from one of the participants at the first time point. Bold indicates the presence of a gesture.

<table>
<tr><td>Catalan</td><td>Hi havia una vegada</td><td>un</td><td>ratolí</td><td>que</td><td>va</td><td>a</td><td>estendre</td><td>la</td><td>roba</td></tr>
<tr><td>Glosses</td><td>Once upon a time</td><td>indef. pron.</td><td>mouse</td><td>relative pron.</td><td>go-3SG</td><td>prep.</td><td>hang out</td><td>the</td><td>clothes</td></tr>
<tr><td>English</td><td colspan="9">Once upon a time there was a mouse who was going to hang out the washing</td></tr>
<tr><td>Gesture Phase</td><td colspan="5"></td><td colspan="2">stroke</td><td colspan="2"></td></tr>
<tr><td>Gesture Referentiality</td><td colspan="5"></td><td colspan="2">Non-referential</td><td colspan="2"></td></tr>
<tr><td>Target Word</td><td colspan="6"></td><td>Estendre</td><td colspan="2"></td></tr>
<tr><td>Focus / Background</td><td colspan="9">Focus</td></tr>
<tr><td>Topic / Comment</td><td colspan="9">Comment</td></tr>
<tr><td>Referent Status</td><td></td><td colspan="8">r-new</td></tr>
</table>

<table>
<tr><td>Catalan</td><td>I</td><td>feia</td><td>molt de vent.</td><td>anava</td><td>ficant</td><td>la</td><td>roba,</td></tr>
<tr><td>Glosses</td><td>and</td><td>was-SG</td><td>a lot of wind</td><td>when</td><td>put-gerund</td><td>def. pron.</td><td>clothes</td></tr>
<tr><td>English</td><td colspan="7">But it was windy. He was putting the clothes [in the rope],</td></tr>
<tr><td>Gesture Phase</td><td colspan="7"></td></tr>
<tr><td>Gesture Referentiality</td><td colspan="7"></td></tr>
<tr><td>Target Word</td><td colspan="7"></td></tr>
<tr><td>Focus / Background</td><td></td><td colspan="2">Focus</td><td colspan="4">Focus</td></tr>
<tr><td>Topic / Comment</td><td></td><td>Comment</td><td>Aboutness Topic</td><td colspan="4">Comment</td></tr>
<tr><td>Referent Status</td><td colspan="2"></td><td>r-new</td><td colspan="2"></td><td colspan="2">r-given</td></tr>
</table>

Catalan	però	amb	el	vent	li	queia	la	roba
Glosses	but	prep.	def. pron.	wind	3SG	fall down-3SG	def. pron.	clothes
English	but the wind was blowing the clothes down.							
Gesture Phase								
Gesture Referentiality								
Target Word								
Focus / Background	Focus							
Topic / Comment		Frame-setting Topic			Comment		Aboutness Topic	
Referent Status		r-given			r-given		r-given	

Catalan	I	doncs,	va	desfer	una	**corda**
Glosses	and	then	untie-3rd-SG		indef. pron.	rope
English	Thus, he unties a rope.					
Gesture Phase			**stroke**			
Gesture Referentiality			**Referential**			
Target Word						**Corda**
Focus / Background			**Focus**			
Topic / Comment			**Comment**			
Referent Status					**r-accessible**	

Catalan	Va **ficar**	per...	pel	**mig**	i	després	el	vent	ja	no	podia	treure-la
Glosses	put-3SG	prep.	prep. + def. pron.	center	and	then	def. pron.	wind	yet	neg.	can-3 SG	take off-INF.-3SG
English	[He] put [the clothes] inside [the rope] and then the wind could not blow it down.											
Gesture Phase	**stroke**		**stroke**									
Gesture Referentiality	**Referential**		**Referential**									
Target Word	**Ficar**			**Mig**								
Focus / Background	**Focus**						Background		Focus			
Topic / Comment	**Comment**					Frame-setting Topic	Aboutness Topic		Comment			
Referent Status			**r-new**				r-given					r-given
